# Demystifying longitudinal melanonychia: Low-cost 3D-printed nail models for patient education

**DOI:** 10.1016/j.jdin.2026.05.010

**Published:** 2026-05-17

**Authors:** Nuran C. Golbasi, Zachary J.K. Neubauer, NatalieDeana Badillo, Shari R. Lipner

**Affiliations:** a Weill Cornell Medicine, New York, New York; b Sidney Kimmel Medical College, Thomas Jefferson University, Philadelphia, Pennsylvania; c Department of Dermatology, Weill Cornell Medicine, New York, New York

**Keywords:** 3D models, 3D printing, dermatology education, longitudinal melanonychia, nail disorders, patient education

## Challenge

Three-dimensional (3D) models are increasingly being used as educational tools. Prior studies demonstrated that use of 3D models improved patient understanding and experiences during clinical encounters.^1^ For example, a randomized trial of 82 patients who underwent Mohs surgery showed higher knowledge scores with 3D models versus standardized education (93.3% vs 85.8%; *P* < 0.028) and trended toward greater reduction in perioperative anxiety (anxiety score change −1.31 vs −0.52; *P* = 0.052).^2^ However, clinical use of 3D models is constrained by high costs and limited availability of disease-specific models. Nail disorders present a particular challenge, as the complex anatomy and continuous growth of the nail unit are difficult for patients to conceptualize using traditional static diagrams and verbal explanations alone. For example, the etiopathogenesis of longitudinal melanonychia can be difficult to visualize, since pigment originates in the nail matrix but becomes visible later in the nail plate as it grows outward.

## Solution

3D printing offers a practical method to create low-cost, disease-specific educational models using an accessible and reproducible workflow (Supplementary Video 1, available via Mendeley at https://data.mendeley.com/datasets/4j6y6cvj92/2). In collaboration with the school of design, we translated conceptual representations of key nail unit anatomy into digital 3D models using computer-aided design software. The model consists of 2 3D printed components, a nail unit base and a removable nail plate, which fit together directly after printing. Computer-aided design files and printing instructions are provided in Supplementary File 1, available via Mendeley at https://data.mendeley.com/datasets/4j6y6cvj92/2. The models were optionally hand-painted to highlight anatomical features, and a clear sealant was applied to our prototypes to improve surface finish and durability.

**Fig 1:**
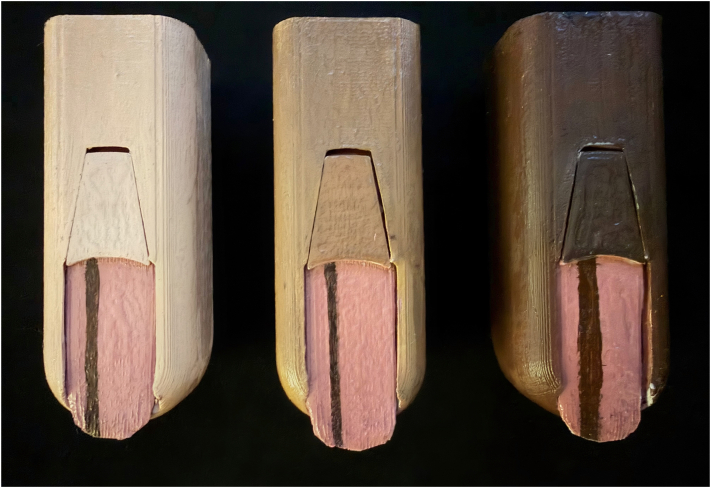
3D-printed nail unit models demonstrating longitudinal melanonychia across Fitzpatrick skin types.

**Fig 2:**
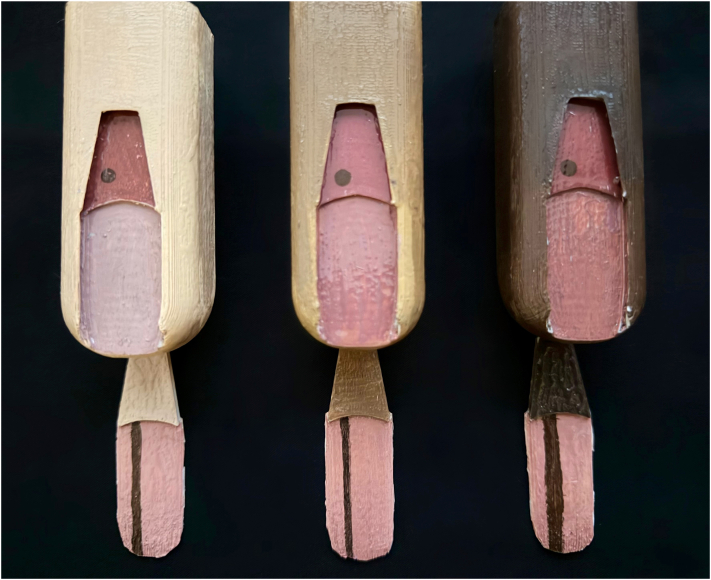
3D-printed nail unit models with nail plate removed demonstrating nail matrix anatomy and pigment origin.

Using this workflow, we produced inexpensive, reusable nail unit models to illustrate the etiopathogenesis of longitudinal melanonychia (Fig 1). The removable nail plate permits stepwise explanation of (1) nail matrix anatomy, (2) localization of pigment in the nail matrix, and (3) incorporation of pigment into the nail plate (Fig 2). To support personalized and inclusive patient education, we created 3 variants corresponding to Fitzpatrick types I-II, III-IV, and V-VI. Total cost of materials was approximately $8.26 per model, enabling low-cost scalability. Importantly, this workflow can be adapted to create models for other nail disorders or procedural education. This approach addresses a key gap in patient education for nail disorders using an inexpensive, customizable, and clinic-ready design.

### Declaration of generative AI and AI-assistedtechnologies in the writing process

AI was not used in the preparation of this work.

## Conflicts of interest

Dr Lipner has served as a consultant for Moberg Pharmaceuticals and BelleTorus Corporation. Authors Neubauer, Golbasi, and Badillo have no conflicts of interest to declare.
